# Supplementary material for: Biosynthesis of Rubellins in Ramularia collo-cygni—Genetic Basis and Pathway Proposition
Source: Int J Mol Sci. 2022 Mar 23;23(7):3475. doi: 10.3390/ijms23073475 (PMC8998751; doi:10.3390/ijms23073475)
Supplement: Supplementary file 1 [file ijms-23-03475-s001.zip › FigureS1.pdf]

Supplementary information

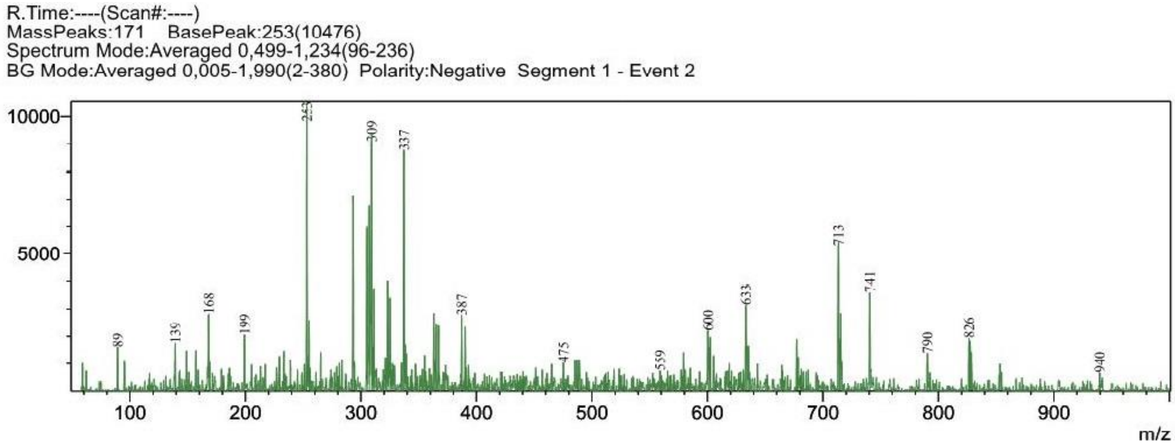

Figure S1. Observed mass of the chrysophanol **7**, the most probable intermediate in rubellin biosynthesis. Molecular ion $[M - H]^-$ = 253.10 was observed in negative ion mode.
